# A reinforcement learning method for optimal control of oil well production using cropped well group samples

**DOI:** 10.1016/j.heliyon.2023.e17919

**Published:** 2023-07-04

**Authors:** Yangyang Ding, Xiang Wang, Xiaopeng Cao, Huifang Hu, Yahui Bu

**Affiliations:** a School of Petroleum and Natural Gas Engineering, Changzhou University, No. 1 Middle Penghu Road, Wujin District, Changzhou, Jiangsu, China; b Shengli Oilfield Exploration and Development Research Institute, Sinopec, No. 2, Liaocheng Road, Dongying District, Dongying, Shandong, China

**Keywords:** Production optimization, Reinforcement learning, Image enhancement, Optimal control, Generalization capability

## Abstract

The influence of geological development factors such as reservoir heterogeneity needs to be comprehensively considered in the determination of oil well production control strategy. In the past, many optimization algorithms are introduced and coupled with numerical simulation for well control problems. However, these methods require a large number of simulations, and the experience of these simulations is not preserved by the algorithm. For each new reservoir, the optimization algorithm needs to start over again. To address the above problems, two reinforcement learning methods are introduced in this research. A personalized Deep Q-Network (DQN) algorithm and a personalized Soft Actor-Critic (SAC)algorithm are designed for optimal control determination of oil wells. The inputs of the algorithms are matrix of reservoir properties, including reservoir saturation, permeability, etc., which can be treated as images. The output is the oil well production strategy. A series of samples are cut from two different reservoirs to form a dataset. Each sample is a square area that takes an oil well at the center, with different permeability and saturation distribution, and different oil-water well patterns. Moreover, all samples are expanded by using image enhancement technology to further increase the number of samples and improve the coverage of the samples to the reservoir conditions. During the training process, two training strategies are investigated for each personalized algorithm. The second strategy uses 4 times more samples than the first strategy. At last, a new set of samples is designed to verify the model’s accuracy and generalization ability. Results show that both the trained DQN and SAC models can learn and store historical experience, and push appropriate control strategies based on reservoir characteristics of new oil wells. The agreement between the optimal control strategy obtained by both algorithms and the global optimal strategy obtained by the exhaustive method is more than 95%. The personalized SAC algorithm shows better performance compared to the personalized DQN algorithm. Compared to the traditional Particle Swarm Optimization (PSO), the personalized models were faster and better at capturing complex patterns and adapting to different geological conditions, making them effective for real-time decision-making and optimizing oil well production strategies. Since a large amount of historical experience has been learned and stored in the algorithm, the proposed method requires only 1 simulation for a new oil well control optimization problem, which showing the superiority in computational efficiency.

## Highlights

• A well control optimization method using reinforcement learning and clipping well group samples is proposed.
• Image enhancement techniques were used to expand the number and coverage of well group samples.
• Two personalized reinforcement learning algorithms for optimizing well production were developed and compared.

## 1 Introduction

The main goal is to maximize the net present value (NPV) or oil production by finding control strategies for each well during reservoir development. The determination of production strategies needs to comprehensively consider the influence of geological development factors such as reservoir heterogeneity, and it is extremely difficult to obtain efficient and precise solutions.

Over the years, numerous algorithms have been developed to tackle production optimization problems. These algorithms can be broadly classified into two categories: gradient-based methods and derivative-free methods [1,2]. Gradient-based optimization methods usually use Adjoint-based approaches to obtain the gradient of the objective function [3,4]. A representative of such methods is gradient-based sequential quadratic programming [5,6]. However, despite their computational efficiency, gradient-based methods may encounter limitations in escaping local minima and reaching the global minimum of the objective function. The derivative-free methods can be divided into approximate gradient algorithms and heuristic algorithms. The commonly used approximate gradient algorithms are Ensemble-Optimization (En-Opt) [7,8] and Simultaneous Perturbation Stochastic Approximation (SPSA) [9]. Approximate gradient algorithms [10,11] can compute approximate numerical gradients instead of using adjoint-based methods. Commonly used heuristic algorithms are Particle Swarm Optimization (PSO) [12], Differential Evolution (DE) [13], and CMA-ES [[14], [15], [16]]. In recent years, heuristic algorithms have attracted considerable attention in the field of production optimization problems. This is due to their remarkable ability to evade local optima, although they often require a large number of function evaluations [17,18]. However, these optimization algorithm-based methods require a large number of iterations, and the experience of these iterations is not preserved by the algorithm. With the advancement of research, there has been a growing trend in proposing hybrid methods to tackle production optimization problems in reservoirs. For example, a hybrid optimization method has been developed that combines sequential quadratic programming (SQP) and ensemble optimization (En-Opt) [19]. In addition, there is the meta-heuristic Bat Algorithm (BA) approach combined with gradient-based Broyden-Fletcher-Goldfarb-Shanno (BFGS) [20].

Over the past few years, remarkable progress has been made in the optimization and control fields, courtesy of reinforcement learning (RL). Notably, RL has emerged as a key contributor in various domains, including robotics, energy control, autonomous driving and games [21,22]. RL is a branch of machine learning where the basic idea is to let the computer learn from the feedback of the environment through continuous experimentation and finally find the optimal strategy. In the petroleum engineering field, RL was initially applied to production optimization problems, specifically for steam injection optimization in thick oil reservoirs [23]. Then, RL was also applied to solve real-time water injection optimization problems [24]. The integration of deep learning into RL, known as deep reinforcement learning (DRL), has shown exceptional performance in handling high-dimensional state-action spaces with an end-to-end perception and decision structure [25]. Various advanced DRL algorithms have also been used by researchers to adjust the injection rate to optimize the NPV of the injection process [26]. However, the learning and training of the above method is performed for the specified model, and the trained model has good applicability to the current reservoir and cannot be applied to other reservoirs. In the realm of field development optimization and reservoir management, recent studies have focused on utilizing RL to address more generic aspects across different reservoir models. For example, researchers combined pixel data using end-to-end strategy optimization to effectively maximize the NPV of the production process [27]. To tackle the challenges associated with scalable field development optimization problems, an innovative artificial intelligence (AI) framework has been developed based on DRL [28,29]. In addition, in the subsurface flow environment, an integrated control strategy framework is introduced to implement closed-loop decision-making using DRL [30]. However, these studies still have some limitations, such as a large number of constraints are required by the model, the model needs millions of times training, and the trained model is difficult to effectively transfer to other reservoirs.

To address the above problems, in this research we no longer take the entire reservoir as a training sample, but take the oil well in the center, and divide the reservoir into many well groups each training sample represents a well group which has different geological and development characteristics. In order to further expand the number and coverage of samples, image enhancement technology is used. As a result, an RL dataset covering more situations is constructed. Two reinforcement learning frameworks, namely the personalized Deep Q-Network (DQN) algorithm and the personalized Soft Actor-Critic (SAC) algorithm, are built. To enhance the applicability of the algorithms, we standardized the range of production parameters of the wells recommended by the algorithms. After that, several algorithm models were obtained after the algorithm was trained using samples. Since the model retains the empirical information of historical optimization, it is expected that the model can provide more accurate and faster support for the recommendation of future control schemes.

The rest of the paper is organized as follows. In Section 2, a production optimization problem is introduced. Then the detailed formulation process of the method is provided in Section 3. In Section 4, the dataset required for this study is described. A test case is studied in Section 5. Finally, the main conclusions are summarized in Section 6.

## 2 Problem statement

### 2.1 Production optimization

The aim of production optimization is to optimize the control strategy of each well to maximize financial profitability or hydrocarbon production [26]. In this work, we choose the NPV of the production process as the objective function to measure the effectiveness of optimization. The calculation of NPV is defined by the following formula [2]:

(1) $$NPV=\sum_{n=1}^{N_{t}}\left\{\left[\sum_{j=1}^{N_{prd}}\left(C_{o}\cdot q_{o,j}^{n}-C_{w}\cdot q_{w,j}^{n}\right)-\sum_{k=1}^{N_{inj}}\left(C_{i}\cdot q_{winj,k}^{n}\right)\right]\frac{\Delta t^{n}}{{\left(1+b\right)}^{t^{n}/365}}\right\}$$

In Eq. (1), the variable $N_{t}$ represents the total number of reservoir simulation steps. $N_{inj}$ and $N_{prd}$ correspond to the total number of injection wells and production wells, respectively. Additionally, $C_{o}$ represents the revenue from oil production, $C_{w}$ denotes the cost of treating produced water, and $C_{i}$ represents the cost of water injection. All these variables are measured in USD/STB (United States Dollars per Stock Tank Barrel). In this study, they are specifically set to 70 USD/STB, 5 USD/STB, and 5 USD/STB, respectively. Furthermore, $q_{o,j}^{n}$ and $q_{w,j}^{n}$ denote the oil production rate and water production rate, respectively, for the j th production well during the n th time step, measured in STB/D (Stock Tank Barrels per Day). $q_{winj,k}^{n}$ represents the water injection rate for the k th injection well during the n th time step, also measured in STB/D. The length of the n th time step is denoted by $\Delta t^{n}$, and $t^{n}$ represents the cumulative time up to the n th time step. Both $\Delta t^{n}$ and $t^{n}$ are measured in days. Finally, b represents the annual discount rate, which is set to 0 in this study.

The injection-production optimization problem can be further divided into a multi-step problem and a one-step problem. In this study, we mainly focus on the one-step problem. In other words, after we use the RL algorithm to obtain a production strategy, we will produce according to the production strategy for a period of time, and the strategy will not be changed during the production process.

### 2.2 RL framework

RL is a branch of machine learning where the basic idea is to let the computer learn from the feedback of the environment through continuous experimentation and finally find the optimal strategy [24,31].

RL is composed of five basic parts: agent, environment, action, state, and reward. The general process of RL is as follows:

(1) The agent observes the current state and selects an action.
(2) The agent executes the action in the environment, while observing the feedback provided by the environment, including the reward and the next state.
(3) Afterwards, the agent updates its own strategy based on the feedback information, in order to make better decisions in the future.
(4) This iterative process continues until a specific termination condition is met or a predefined goal is achieved.

In order to optimize the production strategy for each production well in this paper, we define the oil well as the agent. The action taken by the agent is the adjustment of the bottom-hole pressure (BHP). The state of the oil well is determined by various factors, including the current permeability, saturation, and other geological information. During the study, we utilized a virtual simulation environment where the oil reservoir was simulated using Eclipse, a numerical simulation software dedicated to reservoir analysis. In this environment, the primary objective was to maximize the NPV of the well, which served as the reward function.

**Fig. 1:**
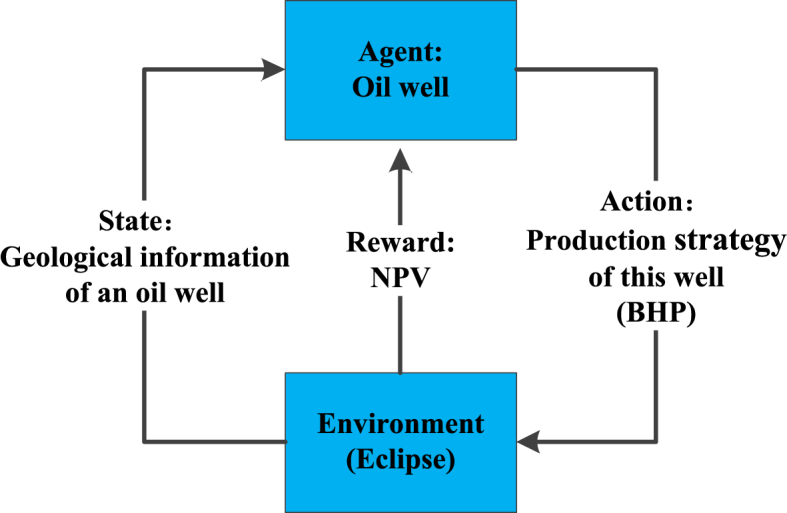
The whole RL process in this study.

Therefore, the entire RL process studied in this paper can be described using Fig. 1. The process for optimizing the production strategy is as follows:

(1) The oil well agent observes the current geological information of the oil well, and chooses a BHP for the well based on its own strategy.
(2) The oil well agent executes the BHP in Eclipse, and observes the feedback from Eclipse, including the NPV and the geological information of the oil well at the next time step.
(3) Next, the oil well agent updates its own strategy based on the feedback information, in order to make better decisions in the future.
(4) Finally, this iterative process continues until a specified termination condition or goal is achieved.

## 3 RL algorithms

### 3.1 Designing personalized RL algorithms

Section 2.2 introduces the basic idea or framework of reinforcement learning. There are many methods for implementing specific algorithms, such as DQN, SAC, and Proximal Policy Optimization (PPO). For the oil well control optimization problem, two personalized reinforcement learning algorithms are designed. One is based on DQN and the other is based on SAC.

Before introducing the two personalized algorithms, it is necessary to provide an explanation of their inputs and outputs to facilitate the understanding of the readers. The inputs and outputs of the two personalized algorithms are presented in Table 1.

**Table 1:** Inputs and outputs of the two personalized algorithms.

Parameter type	Parameter	Simple description
Input	Effective grid field	Each parameter is a 2-dimensional matrix derived from 3-dimensional properties in the reservoir, and each parameter is described in detail in Section 4.1.
	Water saturation field	
	Pressure field	
	Permeability field	
	Pore volume field	
	Injection-production well pattern field	
Output	BHP of the oil well	Production strategy

The inputs and outputs of both algorithms are identical. The inputs consist of six preprocessed reservoir attribute matrices, which can be viewed as six images. The following will be represented by the data of one oil well. The output of the algorithm is the recommended production strategy, i.e., BHP, of the oil well. Based on the aforementioned inputs and outputs, the following two personalized algorithms have been developed.

#### 3.1.1 Designing a personalized DQN algorithm

Basic RL is a value-based optimization method. This method requires evaluating the magnitude of the value corresponding to each action, called the Q-value [32]. But as the state space or action space increases, so does the value of the actions that the method needs to evaluate. This leads to great difficulties in many real-world application problems [33]. For example, Go has 10,170 state spaces, and there is almost no possibility of convergence under the traditional RL paradigm.

To address this limitation, the introduction of Deep Q-learning Network (DQN) [34] provides a solution by utilizing a neural network to approximate the Q-value update process. This kind of neural network is usually called a Q network. The Q network is used to approximate a complex nonlinear function Q(s,a) [35], and the state is mapped to the action value. This method is called the value function approximation method.

To incorporate a neural network for value function approximation, the DQN algorithm introduces two key innovations [34]:

(1) Replay memory: it mainly solves the problems of sample correlation and utilization efficiency. Specifically, during the agent’s interaction with the environment, each step of the experience is stored in replay memory. Data is randomly sampled from the replay memory when the algorithm makes an internal update. Among them, random sampling is to break the correlation existing in a series of observations, and re-sampling the previous data is to improve the utilization rate of the data.
(2) Target network: it mainly solves the problem of unstable algorithm training. Create a two-layer neural network, an offline training network Q with parameter θ, and a target network target Q online with parameter $\bar{\theta }$. Each step of the algorithm will update the parameters of the training network Q, and at each C steps, the parameter θ of the training network is directly assigned to the target network [2].

In neural networks, parameter updating is essentially an optimization problem, that is, minimizing a loss function. Similar to machine learning, the DQN algorithm first defines a loss function and then uses gradient descent to update the parameters.

It is important to note that this study focuses on addressing the single-step optimization problem specific to oil wells. However, it is important to acknowledge that the existing DQN algorithm and the forthcoming SAC algorithm are primarily designed to optimize multi-step time series problems. Therefore, the algorithm has been modified to make it fit our research needs.

In summary, we introduced two key modifications to enhance the DQN algorithm: the network structure of the algorithm and the value formula for calculating actions in the algorithm.

In the general DQN algorithm process [34], it is common to establish a two-layer neural network during initialization, consisting of the original neural network with parameters θ (referred to as the θ network) and the target neural network with parameters $\bar{\theta }$ (referred to as the $\bar{\theta }$ network) [2]. The main goal of incorporating $\bar{\theta }$ networks into the general DQN algorithm is to enhance the stability during network optimization, especially in the case of multi-step time series problems. However, considering the specific objectives of this study, the utilization of the $\bar{\theta }$ network is deemed unnecessary and inconsistent with our research requirements. Therefore, this network needs to be removed.

The value of the current action is calculated by the following expression (2) [2].

(2) $$y_{j}=\left\{ \begin{array}{cc} r_{j} & \text{if}\;\text{episode}\;\text{terminates}\;\text{at}\;\text{step}\;j+1\\ r_{j}+r\;\max_{q^{\prime }}\hat{Q}\left(\varphi_{j+1},a^{\prime };\bar{\theta }\right) & otherwise \end{array}\right.$$

In Eq. (2), $r_{j}$ represents the reward value obtained by performing the current action. The discount rate, denoted r, is a value between 0 and 1 used to discount the value of actions performed in the future to the current time step. $r\;\max_{q^{\prime }}\hat{Q}\left(\varphi_{j+1},a^{\prime };\bar{\theta }\right)$ is an estimate value that evaluates the reward value generated by performing future action $a^{\prime }$ in state $\varphi_{j+1}$. However, considering the specific focus of this study on the single-step optimization problem, where only one action is generated per training iteration without considering $a^{\prime }$, there is no necessity to estimate the $a^{\prime }$. So, there is no need to estimate the value of future actions. Therefore, $\max_{q^{\prime }}\hat{Q}\left(\varphi_{j+1},a^{\prime };\bar{\theta }\right)$ in Eq. (2) needs to be removed. Eventually, Eq. (2) becomes *y*_*j*_ = *r*_*j*_.

In summary, the pseudocode of Algorithm 1 below presents the personalized DQN algorithm designed for this study.

**Table:** 

Algorithm 1:Personalized DQN algorithm
Initialize an empty replay memory *D* with a maximum capacity *N*; Randomly initialize parameters θ to create a starting action-value function *Q*; **For** episode = 1 to *M***do**: Initialize data $s_{1}=\left\{x\right\}$, where x represents the matrix data or image data of an oil well; Get the preprocessed data $\varphi_{1}=\varphi \left(S_{1}\right)$; Get a random number x between 0 and 1 from the computer; IF x <ε then: Randomly choose an action $a_{t}$; Else: The action $a_{t}$ is selected according to the formula $a_{t}={\mathrm{argmax}}_{a}Q\left(\varphi \left(S1\right),a;\theta \right)$; Execute *a*_*t*_ within the reservoir environment; Get the reward $r_{t}$ and $x_{t+1}$; Set $S_{t+1}$ = *S*_*t*_; Get the preprocessed data $\varphi_{t+1}=\varphi \left(S_{t+1}\right)$; Store $\varphi_{t},a_{t},r_{t}{,\varphi }_{t+1}$ in *D* as an experience data; Randomly sample a batch of data $\varphi_{j},a_{j},r_{j}{,\varphi }_{j+1}$ from *D*; Calculate the loss by ${\left(y_{j}-Q\left(\varphi_{j},a_{j};\theta \right)\right)}^{2}$; Update θ using a gradient descent algorithm; End for Output: model with parameter θ.

#### 3.1.2 Designing a personalized SAC algorithm

SAC [36] is a method of continuous control with a random strategy. Actor-Critic in Soft Actor-Critic refers to Actor-Network and Critic Network. Actor-network, also known as Q network, its role is to input state, and then output action. Critic network, also known as strategy network, plays a crucial role in updating its network parameters using historical information and feedback. It then provides guidance for updating the network parameters of the Actor network. Soft in Soft Actor-Critic refers to entropy regularization. The training of the strategy is a trade-off between maximizing expected reward and maximizing entropy. Increasing entropy leads to more exploration. This speeds up later learning and also prevents the strategy from prematurely converging to a local optimum [37,38].

The entropy here refers to the information entropy, which defined as the weighted summation of the information content I(x) associated with all possible values of a random variable x [39]. The expression of information entropy H(x) is as follows:

(3) $$H\left(x\right)=\int_{X}I\left(x\right)dx=\int_{X}-\log\left(P\left(x\right)\right)P\left(x\right)dx$$

P(x) represents the probability of x. Eq. (3) means that the more random the random variable, the greater the entropy.

In general, the goal of RL is to maximize the reward, that is, maximize $Q_{\pi }\left(s,a\right)$. To determine the strategy, we can directly choose the action with the largest $Q_{\pi }$ value to act. But this will make the operating mode hardened. For example, when training a robotic arm to pick up things, the actual trajectory can be varied, and determining the strategy will make this action very monotonous. If it is in a confrontational environment, this fixed operation is also easy to be exploited by opponents and there are loopholes. This can be effectively avoided by executing actions randomly sampled based on strategy π(a|s). The larger the entropy of π, the more random the action, and the more different actions can be made in the same situation, so that the opponent cannot easily predict.

Actor-Critic methods rely on strategy gradients and the goal is to use gradient ascent [38] to maximize $J\left(\theta \right)=E\left[V_{\pi }\left(S\right)\right]$. $V_{\pi }\left(S\right)$ is a function used to measure the value of state S, called the state-value function. Since expectations are not easy to solve, Monte Carlo methods are used to approximate them.

(4) $$\frac{\partial J\left(\theta \right)}{\partial \theta }=E\left[\frac{\partial \log\;\pi \left(A|s;\theta \right)}{\partial \theta }Q_{\pi }\left(s,A\right)\right]\approx \frac{\partial \log\;\pi \left(a|s;\theta \right)}{\partial \theta }Q_{\pi }\left(s,a\right)$$

Assuming that the action a is n-dimensional, then the strategy π uses n Gaussian distributions N(μ,σ) to multiply the way to approximate π. Strategy π can be represented by the following Eq. (5).

(5) $$\pi \left(a|s\right)=\prod_{i=1}^{n}\frac{1}{\sqrt{2\pi }\sigma_{i}}\exp\left(-\frac{{\left(a_{i}-\mu_{i}\right)}^{2}}{2{\sigma_{i}}^{2}}\right)$$

The σ and μ in Eq. (4) are approximated using a neural network [39]. Then the action *a* is sampled according to the obtained n Gaussian distributions. Finally, substitute the action into π in Eq. (4).

SAC adds entropy regularization to the value network. The goal is to further increase the exploratory nature and make the model more robust. This approach is to increase the entropy of the strategy in the reward of each step by modifying the objective function of ordinary strategy learning, so the goal becomes the following Eq. (6).

(6) $$J\left(\theta \right)=E\left[V_{\pi }\left(S\right)+\alpha H\left(\pi \left(\cdot |S\right)\right)\right]$$

It is clear that the network still suffers from overestimation, which is inevitable when maximizing Q values. To solve the overestimation problem, it is necessary to cut off this behavior and the overestimation that results from maximization. SAC avoids the overestimation caused by maximization by using two Q networks and by taking the smallest Q-value. In addition, the strategy entropy balance coefficient α can be manually set with hyperparameters, or it can be adjusted automatically. We do it automatically.

Similar to the DQN algorithm, the SAC algorithm process also needs to be modified to meet the research requirements of this paper. Such as removing the target network and the value estimation part of future actions. The personalized SAC algorithm designed for the oil well parameters is shown in Algorithm 2.

**Table:** 

Algorithm 2: Personalized SAC algorithm
Initialize two *Q* networks with parameters $\theta_{1},\theta_{2}$; Initialize a strategy network with parameters φ; Initialize an empty replay memory *D* with a maximum capacity *N*; **For** episode = 1 to *M***do**: Initialize data $s_{1}=\left\{x\right\}$, where x represents the matrix data or image data of an oil well; Get the preprocessed data $\varphi_{1}=\varphi \left(S_{1}\right)$; According to the strategy network to get action $a_{t}∼\pi_{\varphi }\left(a_{t}|s_{t}\right)$; Execute $a_{t}$ within the reservoir environment; Get the reward $r_{t}$ and $x_{t+1}$; Set $S_{t+1}$ = $S_{t}$; Get the preprocessed data $\varphi_{t+1}=\varphi \left(S_{t+1}\right)$; Store $\varphi_{1},a_{t},r_{t}{,\varphi }_{t+1}$ in *D* as an experience data; Randomly sample a batch of empirical data $\varphi_{j},a_{j},r_{j}{,\varphi }_{j+1}$ from *D*; Update $\theta_{1},\theta_{2}$: $\theta_{i}\leftarrow \theta_{i}-\lambda_{Q}{\hat{\nabla }}_{\theta_{i}}J_{Q}\left(\theta_{i}\right),i\in \left\{1,2\right\}$; Update φ: $\varphi \leftarrow \varphi -\lambda_{\pi }{\hat{\nabla }}_{\varphi }J_{\pi }\left(\varphi \right)$; Update $\alpha :\alpha \leftarrow \alpha -\lambda {\hat{\nabla }}_{\propto }J\left(\propto \right)$; End for Output: model with parameter $\theta_{1},\theta_{2},\varphi$.

### 3.2 Construction of network architecture

#### 3.2.1 Build a personalized DQN network architecture

**Fig. 2:**
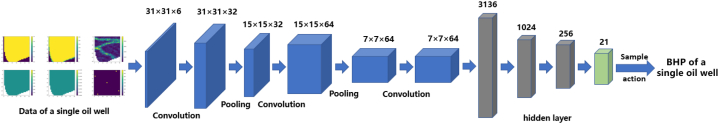
The network architecture of DQN.

The personalized DQN algorithm utilizes a convolutional neural network (CNN) architecture specifically designed for this purpose. The constructed CNN comprises a total of 9 layers, as illustrated in Fig. 2, depicting the network architecture of the DQN model.

The ninth layer is the output layer, and the number of neurons is 21, that is, there are 21 candidate actions. One action corresponds to one BHP, which we will introduce in detail in Section 4.3.

#### 3.2.2 Build a personalized SAC network architecture

**Fig. 3:**
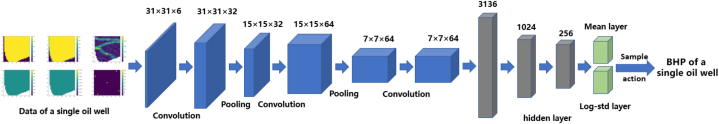
The network architecture of SAC.

The CNN architecture is designed for the personalized SAC algorithm. The constructed neural network has a total of 9 layers. Fig. 3 shows the CNN architecture of the SAC.

The network structure of the first eight layers of the SAC algorithm is the same as that of the DQN algorithm. The ninth layer is the output layer, the number of neurons is 2, one outputs the mean (Mean layer), and the other outputs the variance (Log-std layer). It should be noted that the difference in structure between the output layer of SAC and the output layer of DQN is caused by the fixed structure of the algorithm itself.

## 4 Dataset preparation

### 4.1 Data collection

**Fig. 4:**
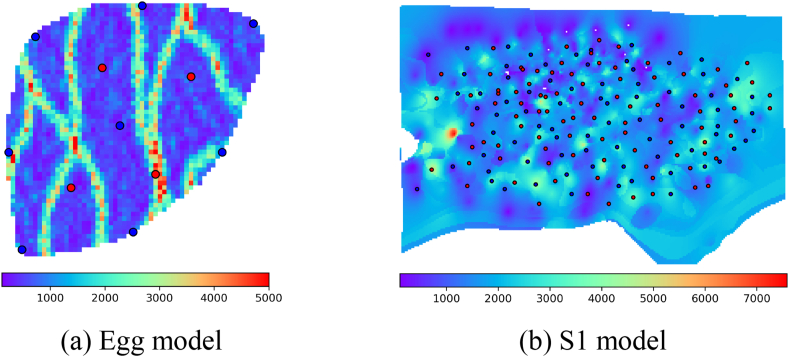
Numerical models of two reservoirs (The red solid circle in the figure represents an oil well, while the blue solid circle represents a water well.). (For interpretation of the references to color in this figure legend, the reader is referred to the Web version of this article.)

The study utilizes the Egg model, which is a widely recognized benchmark model in reservoir production optimization research [40]. At the same time, we built a larger reservoir model of our own, which was named the S1 model. The S1 model is derived from an actual reservoir located in China. The Numerical models of the two reservoirs are shown in Fig. 4. The Egg model comprises grid blocks with dimensions of 60 × 60 × 7, where Δx = Δy = 8 m and Δz = 4 m. In contrast, the S1 model consists of grid blocks with dimensions of 238 × 161 × 1, with Δx = 30.10 m, Δy = 29.44 m, and Δz = 20.60 m. This model is includes 93 oil wells and 77 water wells.

The selection of input parameters is guided by reservoir engineering principles and expert knowledge. The detailed description of each input parameter is as follows.

(1) effective grid field (P1): P1 is used to characterize the geological structure of the reservoir. The dimensions of P1 are relative to the dimensions of the two reservoir models. Each element of P1 can take the value 0 or 1, where 0 means this is an invalid grid, and 1 means this is a valid grid.
(2) pore volume field (P2): P2 is used to reflect the distribution characteristics of the pore volume in the reservoir. The dimensions of P2 are relative to the dimensions of the two reservoir models. The value of each element in P2 is the pore volume of its corresponding grid. Initially, in the Egg model, the pore volume is 51.2 $m^{3}$. For the S1 model, the pore volume ranges from 51.1 $m^{3}$ to 3678.3 $m^{3}$.
(3) permeability field (P3): P3 is used to characterize the permeability distribution of the reservoir. The dimensions of P3 are relative to the dimensions of the two reservoir models. The value of each element in P3 is the permeability of its corresponding grid. For the Egg model, the initial permeability range spans from 81.8 millidarcy to 7000.0 millidarcy. Meanwhile, the S1 model exhibits a permeability range of 100.0 millidarcy to 7581.48 millidarcy.
(4) pressure field (P4): P4 is used to characterize the pressure distribution of the reservoir. The dimensions of P4 are relative to the dimensions of the two reservoir models. The value of each element in P4 is the pressure of its corresponding grid. The Egg has a pressure range of 390.0 bar–406.8 bar and the S1 model has a pressure range of 130.0 bar–210.0 bar.
(5) water saturation field (P5): this parameter is used to characterize the water saturation distribution of the reservoir. The dimensions of P5 are relative to the dimensions of the two reservoir models. The value of each element in P5 is the water saturation of its corresponding grid. The water saturation ranges from 0.1 to 0.81 for the Egg model and from 0.42 to 0.90 for the S1 model.
(6) injection-production well pattern field (P6): P6 is used to characterize the positional relationship between oil and water wells in the reservoir and the control strategy of each well. The dimensions of P6 are relative to the dimensions of the two reservoir models. If a grid has no well perforations, the element corresponding to this grid is 0, otherwise, the element corresponding to the grid is the BHP of the well. In the Egg model, the default pressure settings for oil and water wells are 390 bar and 405 bar, respectively. Additionally, the BHP for each well in the Egg model is allowed to vary within the range of 380 bar–400 bar. Similarly, in the S1 model, the default pressure settings for water and oil wells are 210 bar and 155 bar, respectively. The pressure range of each well is defined between 130 bar and 180 bar.

Further, the 3-dimensional properties are merged into 2-dimensional matrices. According to reservoir engineering theory, different physical properties have different processing strategies. For P1,

(7) $$x_{i,j}=\max\left(\left[x_{i,j,1},\cdots ,x_{i,j,M}\right]\right)$$

For P2, P3, P4, and P5,

(8) $$x_{i,j}=\frac{1}{M}\sum_{k=1}^{M}x_{i,j,k}$$

For P6,

(9) $$x_{i,j}=\frac{\sum_{k=1}^{M}x_{i,j,k}}{\sum_{k=1}^{M}w_{i,j,k}},w_{i,j,k}=\left\{ \begin{array}{cc} 0 & x_{i,j,k}<=0\\ 1 & x_{i,j,k}>0 \end{array}\right.$$

For Eq. (7), Eq. (8), and Eq. (9), $x_{i,j,k}$ corresponds to the property value of each grid under the 3-dimensional reservoir. $x_{i,j}$ is a value in the 2D matrix after properties the 3D attribute to a 2D matrix. i, j, and k represent the coordinates of the three dimensions of x, y, and z on the reservoir, respectively. The range of values for i and j is 1–60, and the range of values for k is 1–7. M is the number of layers within the reservoir, and M = 1 or 7 in this paper.

**Fig. 5:**
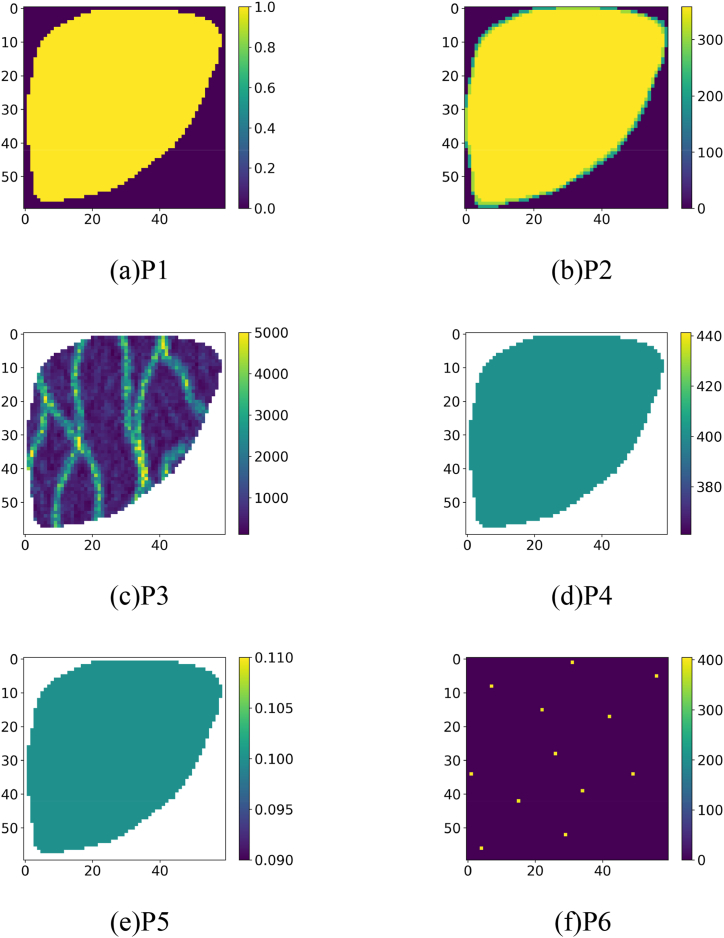
2-dimensional matrix derived from 3-dimensional properties.

After the above processing, each model can get 6 matrix data, or it can also be described as 6 images. Taking the Egg model as an example, the obtained reservoir data are shown in Fig. 5. This figure showcases a set of 2-dimensional matrices originating from the 3-dimensional properties of the reservoir. Specifically, it includes the following components: (a) P1, (b) P2, (c) P3, (d) P4, (e) P5, and (f) P6.

### 4.2 Dataset preparation

The next step is to crop the image around each well. In order to follow reservoir engineering theory, the effective control range of each well determines the size of the well group to be clipped. In the case of the Egg model, the cropping frame size is set to 31 × 31, while for the S1 model, it is 11 × 11. To facilitate subsequent model training, the 11 × 11 size samples are changed to 31 × 31 size samples by interpolation. Specifically, we use a bi-linear interpolation method, which is commonly used for image resizing tasks. Bi-linear interpolation involves computing a weighted average of the four nearest pixels to estimate the value of a new pixel. By applying this interpolation method, we can obtain high-quality images that are suitable for model training. Other interpolation methods, such as bi-cubic interpolation and nearest-neighbor interpolation, were also considered, but were found to produce lower-quality images for our specific application.

**Fig. 6:**
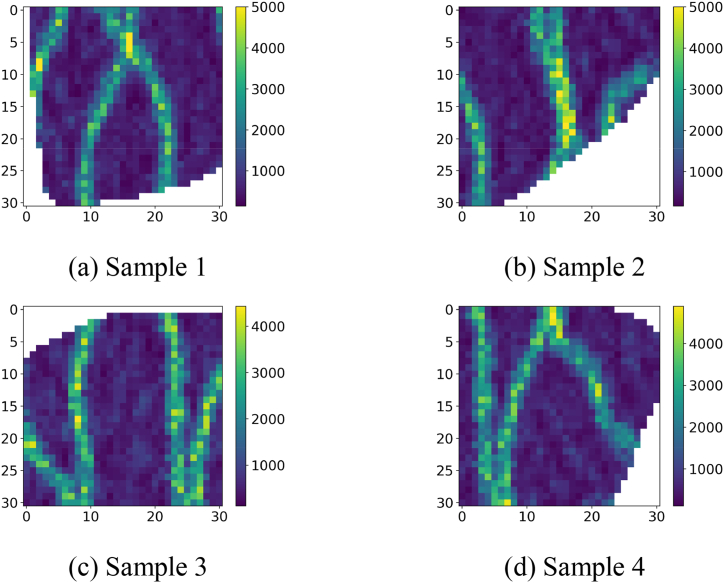
Cropped samples centered on each well in the permeability field of the Egg model.

Since the Egg and S1 models have a total of 97 wells, 97 images of size 31 × 31 were cropped with each well as the center. The four subfigures (a)–(d) in Fig. 6 show the cropping results of the permeability field in the Egg model.

The same cropping operation is taken for 6 input images to obtain 97 sets of samples. Each set of samples contains 6 images of size 31 × 31.

### 4.3 Image enhancement

The purpose of image enhancement is to expand the dataset size through the application of various randomized modifications to the images. This process incorporates a range of common transformations such as rotation, flip, scaling, noise perturbation, color variation, and others. These alterations introduce similar yet distinct samples into the dataset. Additionally, the randomization of sample modifications reduces the model’s reliance on specific attributes, thereby enhancing the model’s generalization capabilities [41].

The purpose of this paper is to optimize the production strategy for each production well. Therefore, the required samples should be related to each well. The generalization ability of the trained RL algorithm model is closely related to the diversity of samples. In other words, the more reservoir geological development conditions covered by the samples used to train the RL algorithm, the higher the generalization and applicability ability of the model. Therefore, in order to make the samples meet the research requirements, it is necessary to perform image enhancement processing on the acquired data. The image enhancement methods used in this study are mainly image rotation and image flipping.

**Fig. 7:**
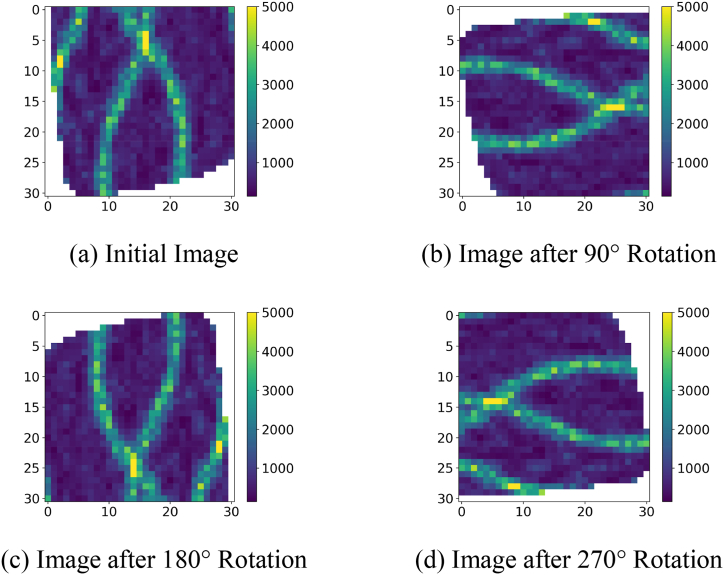
Permeability image rotations.

First, the original image is rotated by 90°, 180° and 360°, resulting in three new images. These images are shown in subfigures (a), (b), (c) and (d) in Fig. 7. For our case study, the initial number of samples can be expanded to four times the original number of samples, i.e., 388 samples, by image rotation.

**Fig. 8:**
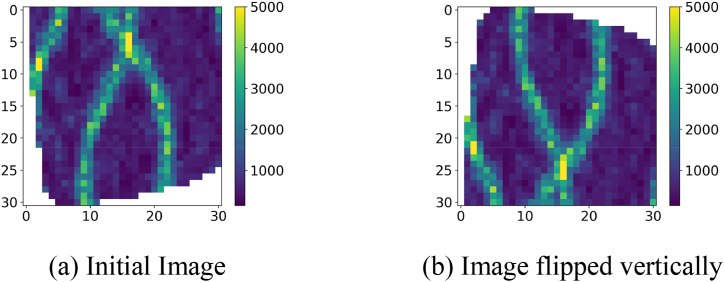
Permeability image vertical flip.

Then there is the image flip, we flip an image vertically and a new image will be obtained, as shown in the subgraphs (a) and (b) of Fig. 8. The sample of 388 wells obtained from the rotation is made to flip vertically to obtain 388 brand new samples again.

In order to train the algorithm and also to check the extent to which image enhancement affects the training effect of the algorithm. We prepared a sample set based partially on the initial 97 samples to train the algorithm, called training set 1. In addition, we prepared a training set 2 for training the algorithm, which includes part of the initial samples as well as part of the samples obtained by image enhancement. And, the number of samples in training set 2 is four times more than the number of samples in training set 1. For the validation set, we randomly selected two sample sets from the initial samples and the samples obtained by image enhancement, which are called validation set 1 and validation set 2, respectively. It is worth noting that the samples in the validation set are not the same as the data in the training set, and that the number of samples in validation set 2 is four times larger than the number of samples in validation set 1.

## 5 Case study

### 5.1 Model training process

To systematically compare and analyze the performance of different algorithms under different datasets, we designed 4 experiments as in Table 2.

**Table 2:** The datasets and algorithms used in the four experiments.

Experiment No.	Dataset	Algorithm
Experiment 1	Training set 1	Personalized DQN
Experiment 2	Training set 2	Personalized DQN
Experiment 3	Training set 1	Personalized SAC
Experiment 4	Training set 2	Personalized SAC

**Fig. 9:**
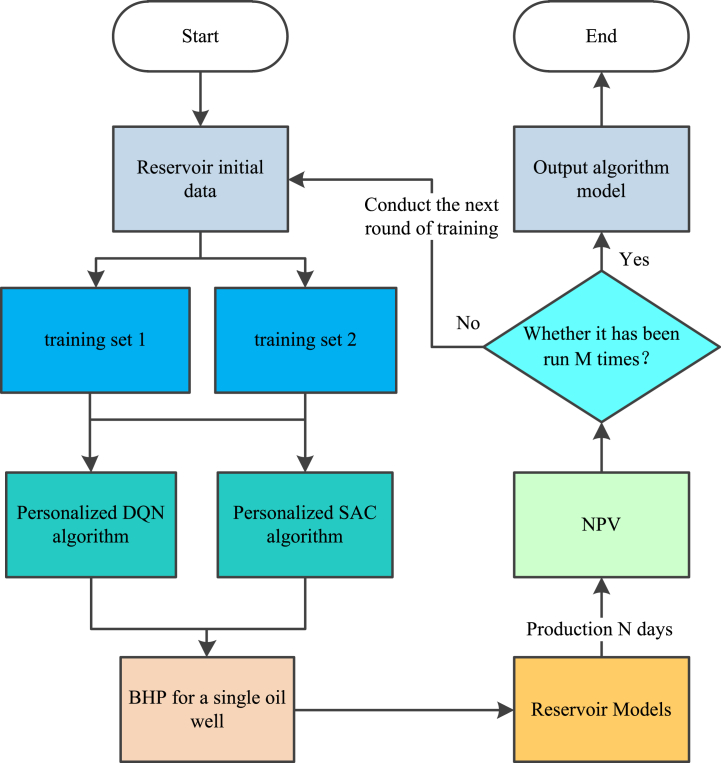
Algorithm training process.

The algorithm training process in each experiment is shown in Fig. 9. At the start of the algorithm, the reservoir data is first initialized. Then, according to the current experiment, the corresponding training dataset is inputted into the personalized algorithm. Next, the personalized algorithm outputs the BHP of the oil well based on the input data. Then, the oil well will produce for N days (in this paper, 1080 days) under the given production strategy. After that, the algorithm can obtain the NPV of the oil well. At this point, after each NPV calculation, the algorithm checks if it has run for more than the maximum number of times M (in this paper, 20,000 times). If the algorithm has been trained for M times, it will be terminated and the trained model will be saved. Otherwise, the algorithm will continue to run until the termination condition is met.

Several points need to be explained below.

(1) In Section 4.1, we introduced the constraints for oil wells and water wells. To enhance the applicability of the algorithm, we set the pressure adjustment range for each oil well to [−1, 1]. To reduce the difficulty of model training, we discretize the values within this range into 21 values, with an interval of 0.1. The value 0 within this range corresponds to the initial pressure of each oil well, and the range boundaries correspond to the upper and lower limits of BHP for each well. Therefore, each well will have 21 pressure candidates, i.e., 21 different actions. These 21 actions correspond to the output layer neurons in the algorithm structure in Section 3.2.1.
(2) We use million US dollars (MUSD) as the unit for NPV calculation and keep two decimal places. In our experiments, it has been proven that doing so can make the algorithm training process more stable and converge faster.
(3) According to the pseudocode for the DQN and SAC algorithms, both algorithms need to optimize their network parameters by extracting a batch of experience data from the replay memory. However, at the initial stage of the algorithm’s operation, the algorithm only produces one experience per run, and the amount of experience data is insufficient for algorithm training. Therefore, the replay memory needs to store some data in advance. In this study, the data generated by the algorithm in the first 10,000 runs will be stored in the replay memory. During this period, the network will not be updated. This will also allow the algorithm to fully explore and help accelerate the convergence speed of the algorithm. After replay memory stores 10,000 data, the algorithm evaluates the network performance every 100 runs and records the training results.

### 5.2 Model training results and analysis

The results of each experiment will be presented using two subplots, namely subplot (a) and subplot (b). Subplot (a) shows the change in the target optimal NPV during training, while subplot (b) shows the change in the target recommended best bottom hole pressure during training. All images in subplot (a) share the same horizontal axis at the bottom row. All images in subplot (b) share the same vertical axis on the left column and the same horizontal axis at the bottom row. The algorithm is run 100 times, referred to as 1 episode.

#### 5.2.1 Experiment 1

**Fig. 10:**
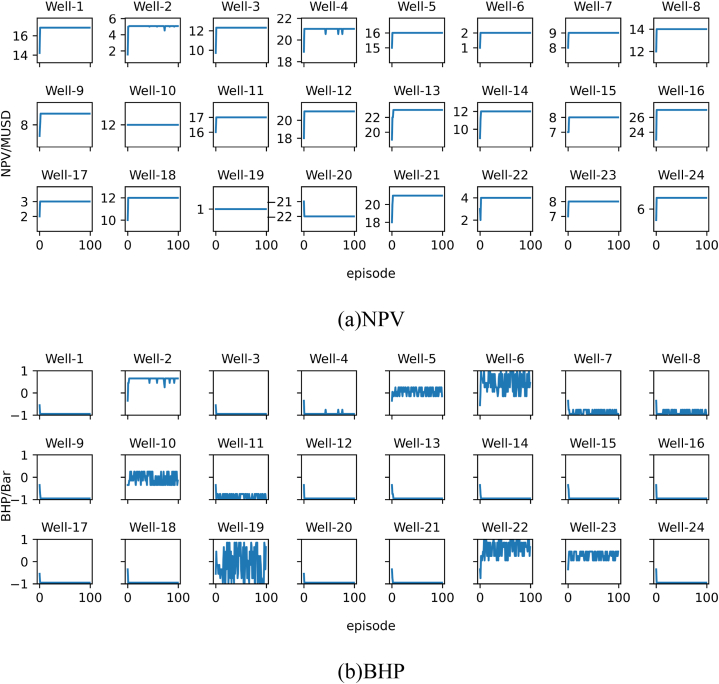
Training results of Experiment 1. (a) is the NPV record of each well, and (b) is the BHP record corresponding to the NPV of each well in (a). The Well-n in the subfigure represents the well number.

The results of Experiment 1 are presented in Fig. 10. From Fig. 10a we can see that, if the personalized DQN is trained using training set 1, all wells can converge within three episodes. Fig. 10b shows the optimal control strategy recommended by the personalized DQN algorithm. Most of the curves in Fig. 10b fluctuate throughout the training process. This indicates although the model appears to converge, it still cannot give a stable solution for many wells. This is because some wells can get same or very close NPV under different BHPs. In other words, the optimal BHP for some wells has multiple solutions. Hence the output of the personalized DQN algorithm jumps between several optimal BHPs. To prove our hypothesis, we calculated the NPV of each well at each BHP level. The results confirm the above situation.

#### 5.2.2 Experiment 2

**Fig. 11:**
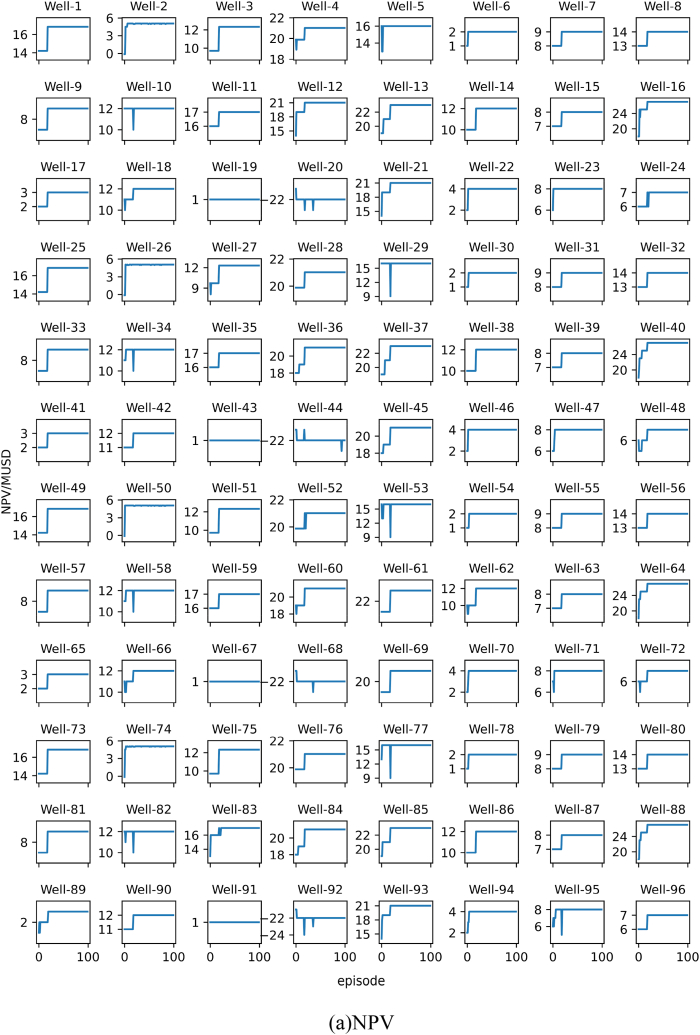

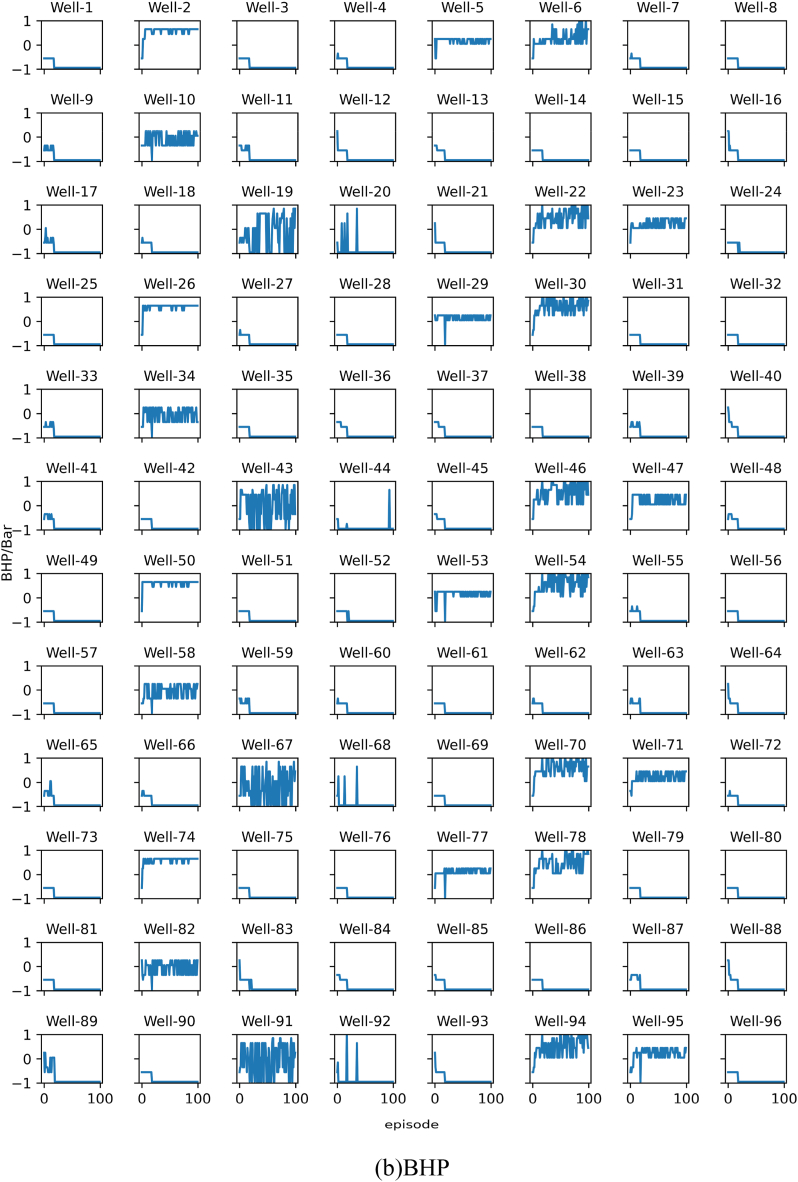
Training results of Experiment 2.

The results of Experiment 2 are presented in Fig. 11. Fig. 11 shows the training results of the personalized DQN using training set 2. Since training set 2 has more samples, the convergence time in Fig. 11a is slower compared to the results in Fig. 10a. Similarly, the fluctuate phenomenon shown in Fig. 10b also appears in Fig. 11b.

#### 5.2.3 Experiment 3

**Fig. 12:**
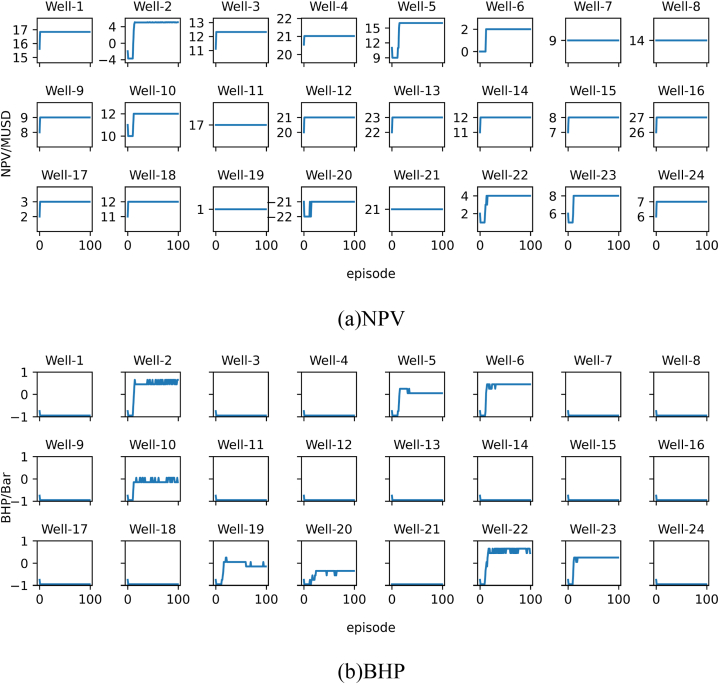
Training results of Experiment 3.

The results of Experiment 3 are presented in Fig. 12. The experiment replaced the DQN algorithm with the SAC algorithm in Experiment 1. As shown in Fig. 12a, the SAC algorithm also achieved convergence. Similarly, the curve in Fig. 12b also fluctuates, but compared with the curve in Fig. 10b, the amplitude of the fluctuation in the experimental result is smaller.

#### 5.2.4 Experiment 4

**Fig. 13:**
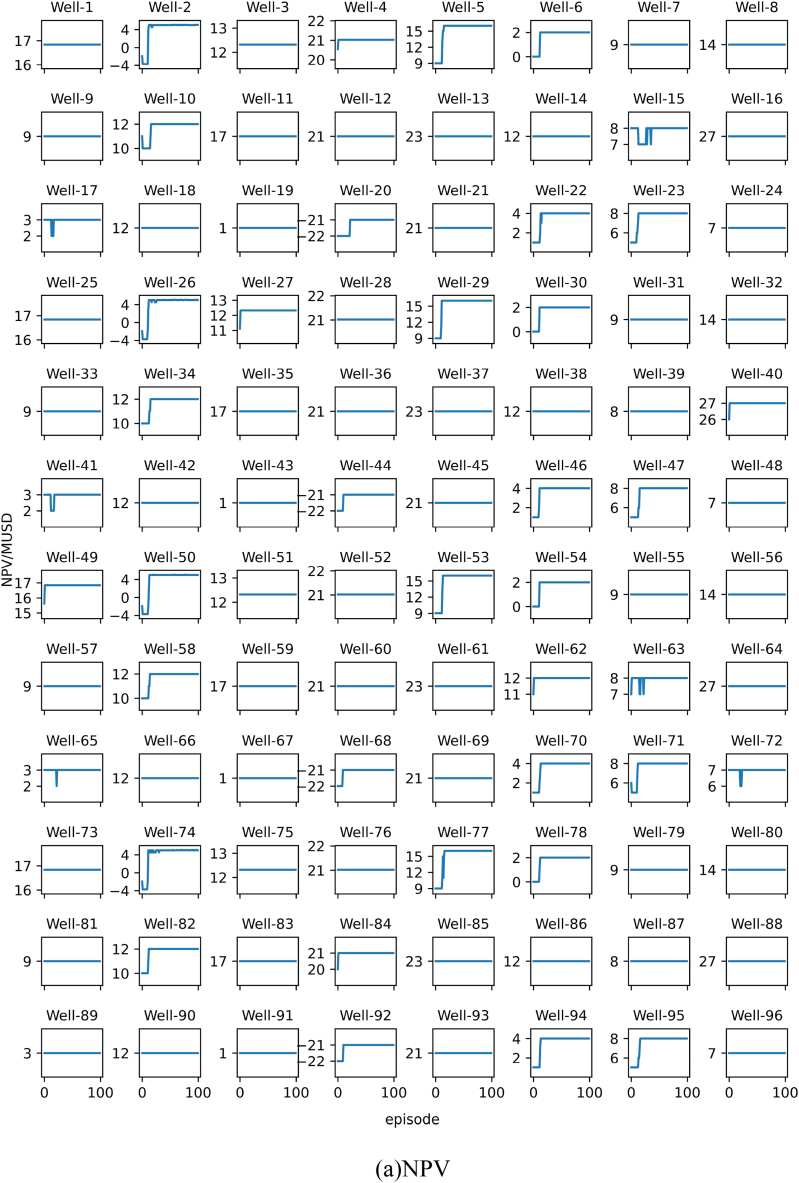

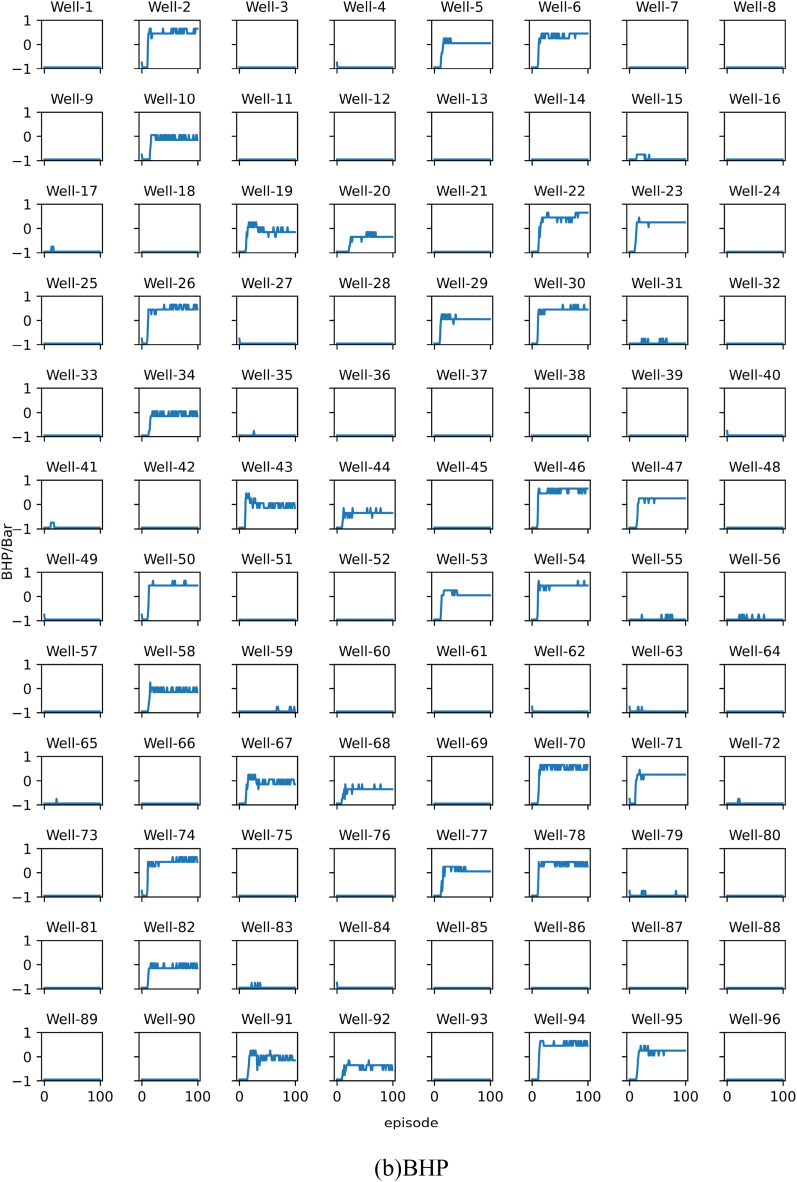
Training results of Experiment 4. In summary, the personalized DQN algorithm converges faster than the personalized SAC algorithm, but the stability of the personalized SAC algorithm is better than the former.

The results of Experiment 4 are presented in Fig. 13. The experiment replaced the DQN algorithm with the SAC algorithm in Experiment 2. As shown in Fig. 13a, the SAC algorithm also achieved convergence. Similarly, the curve in Fig. 13b also fluctuates, but compared with the curve in Fig. 11b, the amplitude of the fluctuation in the experimental result is smaller.

### 5.3 Model validation process

During the model validation phase, we designed four validation experiments. Specifically, validation experiment 1 refers to validating the trained personalized DQN model with validation set 1, and validation experiment 2 refers to validating the same model with validation set 2. Validation experiment 3 refers to validating the trained personalized SAC model with validation set 1, and validation experiment 4 refers to validating the same model with validation set 2.

**Fig. 14:**
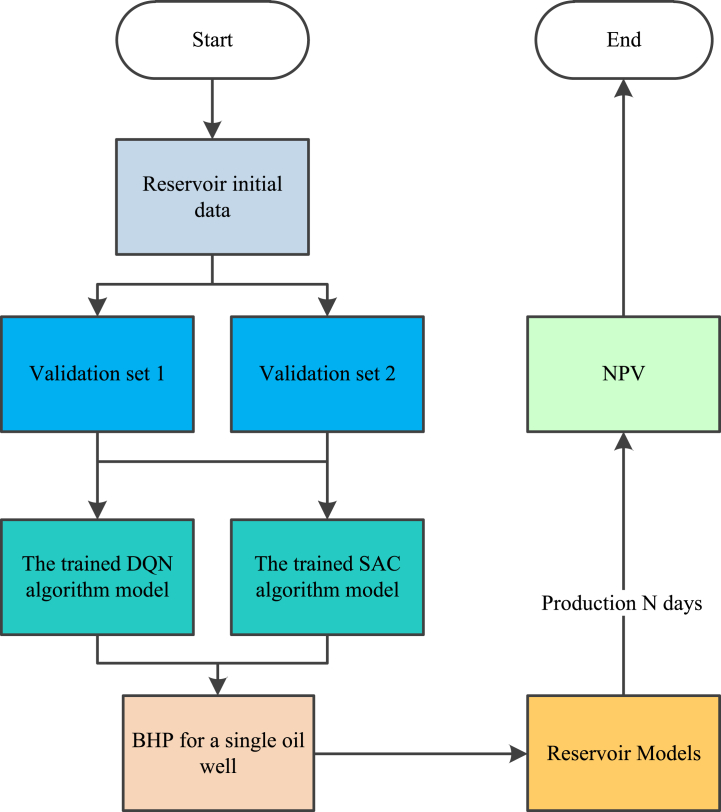
Model validation flow.

The process of each model validation experiment is illustrated in Fig. 14. First, the program initializes the data of the reservoir. Then, according to the current experimental content, the corresponding validation dataset is inputted into the corresponding personalized model. Subsequently, the model outputs the BHP of the oil well based on the input data. Next, the oil well produces for N days according to the given production strategy. Afterwards, the model obtains the NPV of the oil well. Finally, the program stops running.

As can be seen from Fig. 9, Fig. 14, compared to the training process, the validation process only requires one reservoir simulation.

### 5.4 Model validation results and analysis

For each validation experiment, we will present the results using two subplots, namely subplot (a) and subplot (b). Subplot (a) represents the NPV corresponding to the recommended BHP for the oil wells by the model, while subplot (b) represents the recommended BHP for the oil wells by the model.

#### 5.4.1 Validation experiment 1

**Fig. 15:**
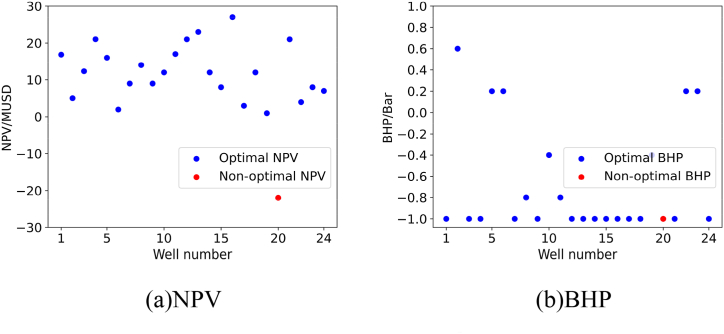
Results of Verification experiment 1. The blue solid circle in the two subplots represents the optimal NPV or BHP recommended by the model, while the red solid circle represents the non-optimal NPV or BHP that the model did not recommend. (For interpretation of the references to color in this figure legend, the reader is referred to the Web version of this article.)

To verify the quality of the model’s recommended solutions, we calculated the NPV of the 24 oil wells used in the validation experiment at different BHP levels and found the global optimal solution. The comparison results between the solution recommended by the model and the global optimal solution are shown in subplot (a) and subplot (b) in Fig. 15.

As shown in the figure, the personalized DQN model recommended the optimal BHP for 23 out of the 24 wells. This indicates that the model performs well when dealing with a limited number of new samples.

#### 5.4.2 Validation experiment 2

**Fig. 16:**
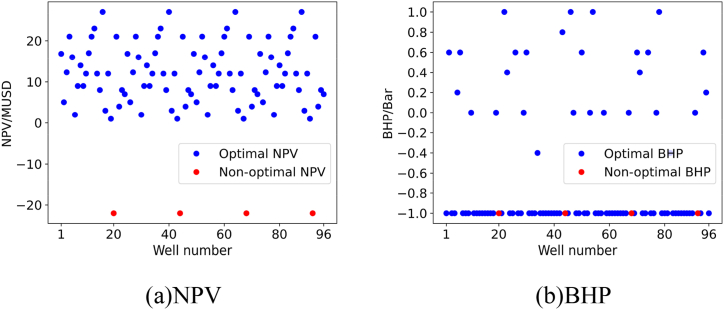
Results of Verification experiment 2.

To validate the quality of the personalized DQN model in recommending control strategies for a larger set of oil wells, we calculated the NPV of 96 oil wells at different BHP levels in validation experiment 2, and found the global optimal solution. The comparison between the recommended solution of the model and the global optimal solution is shown in subplot (a) and subplot (b) in Fig. 16.

From the figure, we can see that the personalized DQN model recommended the optimal BHP for 92 out of 96 oil wells, demonstrating its effectiveness in handling multiple unseen oil well samples.

#### 5.4.3 Validation experiment 3

**Fig. 17:**
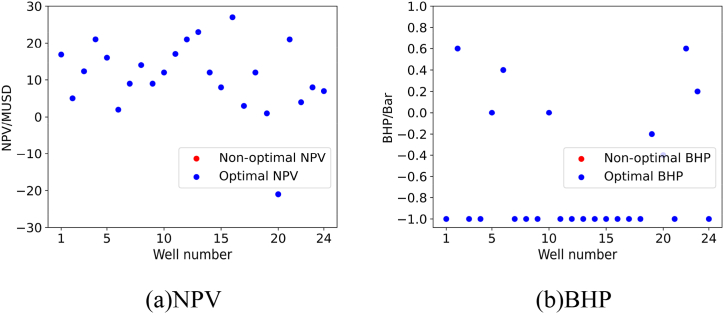
Results of Verification experiment 3.

The model used in this validation experiment was replaced by personalized SAC, which was previously personalized DQN in validation experiment 1. The comparison between the recommended solution of the model and the global optimal solution is shown in subplot (a) and subplot (b) in Fig. 17.

From the figure, it can be seen that the personalized SAC model recommended the optimal BHP for all 24 oil wells. This indicates that when recommending the optimal production strategy for a small number of oil well samples, the personalized SAC model performs better than the personalized DQN model.

#### 5.4.4 Validation experiment 4

**Fig. 18:**
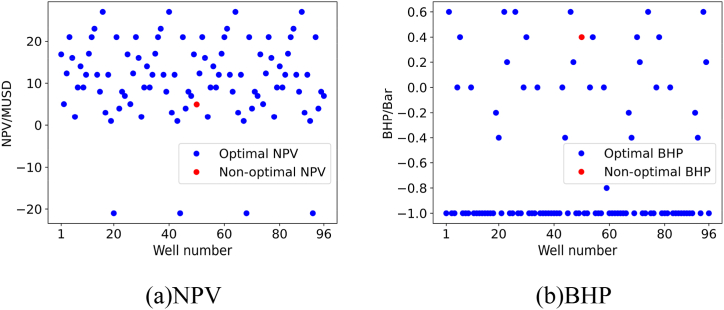
Results of Verification experiment 4.

The model used in this validation experiment was replaced by personalized SAC, which was previously personalized DQN in validation experiment 2. The comparison between the recommended solution of the model and the global optimal solution is shown in subplot (a) and subplot (b) in Fig. 18.

From the figure, we can see that the personalized SAC model recommended the optimal BHP for 95 out of 96 oil wells. This indicates that the personalized SAC model is still superior to the personalized DQN model in recommending the optimal production strategy for the same number of oil well samples.

A comparison between the solutions recommend by the trained model and the global optima is summarized in Table 3. From the four validation results we can see that, for a well sample that has never participated in training process, the trained model still has more than 95% probability of pushing the global optimal control strategy for it. To evaluate the effectiveness of each validation experiment, we define accuracy as the ratio of the number of wells that achieve the optimal solution using the trained model to the total number of wells. This metric serves to demonstrate the efficacy of the proposed approach.

**Table 3:** Validation experiment result statistics table.

Experiment No.	Dataset	Algorithm	Accuracy
Experiment 1	Validation set 1	Personalized DQN	95.83%
Experiment 2	Validation set 2	Personalized DQN	95.83%
Experiment 3	Validation set 1	Personalized SAC	100%
Experiment 4	Validation set 1	Personalized SAC	98.96%

The personalized SAC model performs a better than the personalized DQN model in recommend accuracy. Nevertheless, it should be noted that both models were run only once to solve the optimal control problem for a new well, which shows the superiority of computational efficiency.

### 5.5 Comparative evaluation and results

In this section, we compare the performance of our trained personalized DQN and personalized SAC models with the PSO algorithm in recommending production strategies for four oil wells in the Egg reservoir model. Our objective is to analyze the impact of our proposed models and evaluate their effectiveness in optimizing oil well production.

Based on the research problem and commonly used parameter settings in PSO examples, we set the main parameters for PSO as follows: the NPV obtained from numerical simulation software is used as the fitness function and BHP is used as the independent variable. The algorithm stops after 100 iterations. To explore the range of standardized BHP values, we used 50 particles with a maximum velocity of 0.5 and an inertia factor of 1.0. Additionally, both the social learning factor and the individual learning factor of the particles are set to 2.

In order to provide a comprehensive evaluation, we compared the accuracy and computational efficiency of the three algorithms. The definition of accuracy is the same as in Table 3. Computational efficiency is measured by the average convergence time of the algorithm to optimize the production strategy for four wells. The average convergence time is referred to as the average optimization time in the following text. It should be noted that each algorithm optimizes the BHP for one oil well at a time, so each algorithm needs to run four times. The results are summarized in Table 4.

**Table 4:** Comparative analysis of algorithm performance.

Algorithm	Accuracy	Average Optimization Time (s)
Personalized DQN	100%	<1
Personalized SAC	100%	<1
PSO	100%	1495

From the table, it can be observed that all three algorithms achieved a perfect accuracy of 100% in recommending the optimal production strategies for the Egg reservoir. However, there are significant differences in their computational efficiency.

The personalized DQN and personalized SAC models can optimize the BHP for an oil well in less than 1 s. In contrast, the average optimization time for the PSO algorithm is much longer, at 1495 s.

The longer runtime of the PSO algorithm can be attributed to the nature of its optimization process. PSO generates production strategies that need to be input into Eclipse to evaluate its effect. The simulator requires substantial computational resources and time to complete the simulation, resulting in higher computational costs for the PSO algorithm.

On the other hand, our personalized DQN and personalized SAC models utilize deep neural networks and reinforcement learning techniques to directly acquire and predict the most effective production strategies without relying on evaluations based on simulations. This inherent advantage allows our models to achieve faster computation times, thereby enhancing their efficiency for real-time or near real-time applications.

Furthermore, the ability of our personalized DQN and personalized SAC models to capture complex patterns and adapt to different geological conditions further contributes to their outstanding performance. By training on historical data and continuously updating their knowledge, these models can dynamically adjust production strategies based on new information.

In conclusion, the comparative evaluation demonstrates the computational efficiency advantage of our personalized DQN and personalized SAC models while achieving a perfect accuracy of 100%. Their faster runtime, coupled with their ability to capture complex patterns and adapt to adapt to different geological conditions, makes them highly effective in recommending the optimal production strategies for the Egg reservoir. In contrast, the longer runtime of the PSO algorithm, due to its simulation-based evaluation, highlights the advantages of our personalized models in terms of computational efficiency and real-time decision-making capability.

## 6 Conclusion

In this paper, a personalized DQN algorithm and a personalized SAC algorithm were designed for well control optimization problems, and a corresponding built-in network structure were constructed. The input of the algorithm is the geological information of a well group. The output is the production strategy of the oil well.

A series of well-centered samples were cropped from the whole reservoir. Each sample is a square area that takes an oil well at the center, with different permeability and saturation distribution, and different oil-water well patterns. In addition, to increase the coverage of the samples to the reservoir conditions, all samples are extended by image enhancement techniques.

During the training process, two training strategies are investigated for each personalized algorithm. The second strategy uses 4 times more samples than the first strategy. A new set of samples is designed to verify the model’s accuracy and generalization ability. The results show that the agreement between the optimal control strategies obtained by the two algorithms and the global optimal strategy obtained by the exhaustive method is over 95%. Both the trained DQN and SAC can learn and store historical experience and push appropriate control strategies based on the reservoir characteristics of new wells. The personalized SAC model performs better than the personalized DQN model in terms of recommendation accuracy. Moreover, the method solves the new well control optimization problem with only one simulation, which shows the superiority of computational efficiency.

In addition, we conducted a comprehensive performance evaluation of the two personalized models in comparison with the PSO algorithm. The results demonstrate that both the personalized DQN and personalized SAC models exhibit remarkable computational efficiency advantages over the PSO algorithm, while maintaining comparable accuracy rates.

There are several issues that need to be considered. Future work will focus on improvements, including the addition of vertical geological information from 3D reservoirs to the training samples to enhance the applicability of the trained model to 3D models. Additionally, we acknowledge that further improvements in model accuracy could potentially be achieved through hyperparameter tuning. However, due to time constraints and the limitations of the paper’s length, we did not extensively discuss hyperparameter optimization in this study. Therefore, in future research, we will also devote efforts to investigating the impact of hyperparameter adjustments on the two models.

## Declaration of competing interest

The authors declare that they have no known competing financial interests or personal relationships that could have appeared to influence the work reported in this paper
